# Kinetics and thermodynamics of hydrolysis of crystal violet at ambient and below ambient temperatures

**DOI:** 10.1038/s41598-020-78937-4

**Published:** 2020-12-14

**Authors:** Nurudeen Salahudeen, Adamu A. Rasheed

**Affiliations:** Department of Chemical and Petroleum Engineering, Bayero University, Kano, Nigeria

**Keywords:** Environmental sciences, Chemistry

## Abstract

Hydrolysis reaction was carried out at varying NaOH concentrations of 0.008, 0.016 and 0.024 M, variable temperature of 6 and 21 °C, and constant initial crystal violet (CV) concentration of 2.6 × 10^–5^ M. Kinetic data of the reaction were generated using UV–Vis Spectrophotometer. Analysis of the reaction kinetics shows that the overall rate order of the hydrolysis reaction was 1st order. The individual rate order of the reaction with respect to NaOH and CV was temperature dependent. At 21 °C the rate order with respect to NaOH and CV were 0.24th and 0.76th, respectively. While at 6 °C the individual rate order were 0.38th and 0.62th with respect to NaOH and CV, respectively. Values of the reaction rate constant (k) at 21 and 6 °C were 7.2 and 1.9 $\left( {\frac{{{\text{mol}}}}{{\text{L}}}} \right)^{{ - 0.9}} min^{-1}$, respectively. The activation energy of the reaction was determined as 60.57 kJ/mol. The reaction was an endothermic reaction having enthalpy values of 58.13 and 58.29 kJ/mol at 21 and 6 °C, respectively. The entropy and Gibbs free energy of the hydrolysis reaction at ambient temperature of 21 °C were − 64.72 J/mol K and 77.15 kJ/K, respectively. At 6 °C the entropy and Gibbs free energy of the reaction were − 64.29 J/mol K and 76.19 kJ/K, respectively.

## Introduction

Crystal violet (CV) is a cationic triphenyl methane dye chemically known as hexamethyl pararosaniline chloride. It is a highly demanded industrial raw material used in far-reaching industrial and medical applications. It is used as dye; in textile materials such as cotton and silk, in ternary materials such as leather and animal skin. It is used as an external disinfectant due to its toxic effect on cells. In medical and veterinary applications, it is used as the active ingredient in Gram’s Stain, employed in classifying bacterial^1,2^.

Despite its huge economic benefits, crystal violet constitutes a major environmental threat as it is a key constituent of the waste water from the textile, ternary, and plastic industries. It has also been reported that uncontrolled exposure to crystal violet poses genotoxic and carcinogenic effects on humans and aquatic lives^3,4^. Hence, the need for effective treatment of CV in industrial waste water before disposal cannot be over emphasized. Removal of colour is the basic most important objective of water treatment process in industrial processes where large quantity of recycled water is needed for operations where soluble organic impurities have no impact. Such industrial processes include plastics, leather, paint, textile, acrylic etc.

The hydrolysis of CV can be expressed as Eq. (1). Progress of the reaction can be physically monitored as the deep violet CV stock solution gradually losses its colour as the reaction progresses. At the completion of the reaction the CV solution becomes colourless

1 $${\text{C}}_{{{25}}} {\text{H}}_{{{3}0}} {\text{N}}_{{3}} {\text{Cl}}_{{({\text{aq}})}} + {\text{NaOH}}_{{({\text{aq}})}} \to {\text{C}}_{{{25}}} {\text{H}}_{{{31}}} {\text{ON}}_{{{3}({\text{aq}})}} + {\text{NaCl}}_{{({\text{aq}})}}$$

**Figure 1:**
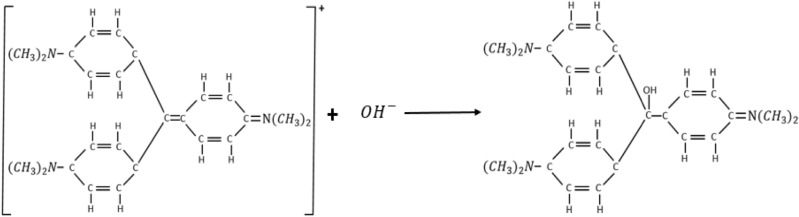
Stoichiometry of the reaction between crystal violet and NaOH^1^.

The crystal violet colour is due to the extensive system of alternating single and double bonds which extends over the three carboxylic rings and the central carbon atom in the CV’s chemical structure. This phenomenon is termed conjugational conformation. Molecules exhibiting extensive conjugational conformation are usually highly coloured. The hydrolysis of CV results into structural stabilization, in the product formed thereby eliminating the conjugational conformation. The resulting product is colourless as the three carboxylic rings are no longer in conjugation^1^. The stoichiometry of the complete reaction is as shown in Fig. 1.

For the hydrolysis reaction in Eq. (1), it follows that

2 $${\text{Rate}} = {\text{k}}\left[ {{\text{CV}}^{{\text{x}}} } \right]\left[ {{\text{OH}}^{ - } } \right]^{{\text{m}}}$$

3 $${-r}_{A}= \frac{-dc}{dt}=k{C}_{A}^{x}{[{OH}^{-}]}^{m}$$

where ${-r}_{A}$ is the rate of reaction with respect to the reactants, *k* is the rate constant of the reaction, ${C}_{A}$ is [CV], the concentration of the crystal violet, x is the order of the reaction with respect to CV, m is the order of the reaction with respect to NaOH, [OH^−^] is concentration of NaOH.

In this experiment, the initial [OH^−^] is made much greater than the initial [CV].

Therefore, since [OH^−^] ≫ [CV], [CV] is the rate limiting reactant as the change in [OH^−^] value during the reaction is negligible. Hence, Eq. (3) can be written as Eq. (3a).

3a $$- r_{A} = \frac{ - dC}{{dt}} = k^{\prime}C_{A}^{n}$$

4 $${\text{where}}\quad k^{\prime} = {\text{k}}\left[ {{\text{OH}}^{ - } } \right]^{{\text{m}}}$$

n is the overall order of the reaction, *k*′ is *pseudo rate constant.* Therefore, the actual rate constant is expressed as Eq. (5).

5 $${\text{k}} = \frac{{k^{\prime}}}{{\left[ {OH^{ - } } \right]^{m} }}$$

For two set of experiments carried out at different values of [OH^−^], it follows that two set of equations hold

6 $$k_{1}^{\prime } = k\left[ {OH^{ - } } \right]_{1}^{m} \;{\text{and}}\;k_{2}^{\prime } = k\left[ {OH^{ - } } \right]_{2}^{m}$$

7 $$\frac{{k_{1}^{\prime } }}{{k_{2}^{\prime } }} = \frac{{{\text{k }}\left[ {{\text{OH}}^{ - } } \right]_{1} {^{m}}}}{{{\text{k }}\left[ {{\text{OH}}^{ - } } \right]_{2} {^{m}}}}$$

8 $$m = \frac{{log\frac{{k_{1}^{\prime } }}{{k_{2}^{\prime } }}}}{{log\frac{{\left[ {{\text{OH}}^{ - } } \right]_{1} { }}}{{\left[ {{\text{OH}}^{ - } } \right]_{2} }}}}$$

Literature information on the activation energy of hydrolysis of crystal violet are scanty, although, some researchers have reported some information about the reaction of crystal violet with sodium hydroxide^5–9^, the depths of most of the reports are not sufficient to give extensive information on the kinetics and thermodynamics information of the hydrolysis reaction. This work is aimed at determining the kinetic and thermodynamic parameters of hydrolysis of crystal violet at ambient and below ambient temperatures. The novel of this study is that unlike other reported works^5–9^ it provides detail kinetic parameters of hydrolysis reaction of crystal violet such as the rate constant, order of the reaction and the rate law. This study also provides the thermodynamic parameters of hydrolysis reaction of crystal violet such as the enthalpy, the activation energy and the entropy at ambient and below ambient temperatures.

## Materials and methods

### Materials

The materials used include; analytic grade crystal violet (Sigma Aldrich, Darmstardt, Germany), analytic grade sodium hydroxide pellets (98% Loba Chemie, Mumbai, India). Distilled water was produced using water distiller (SZ-96, Mon Scientific). Other apparatuses used are stop watch and glassware.

### Calibration curve

A stock solution of crystal violet was prepared by weighting 0.1 g of the powder using weighing balance (Ohaus SP202 Scoutt Pro) and dissolved in 1000 mL of distilled water. Six (6) samples containing 0, 2, 4, 6, 8 and 10 mg/L of CV were prepared by diluting the stock solution with appropriate volumes of distilled water. The absorbance of each of the six (6) samples was measured with a UV–Vis Spectrophotometer (Zuzi; Model 4201/20, France) at a wavelength of 565 nm.

### Reaction kinetics

**Plate 1:**
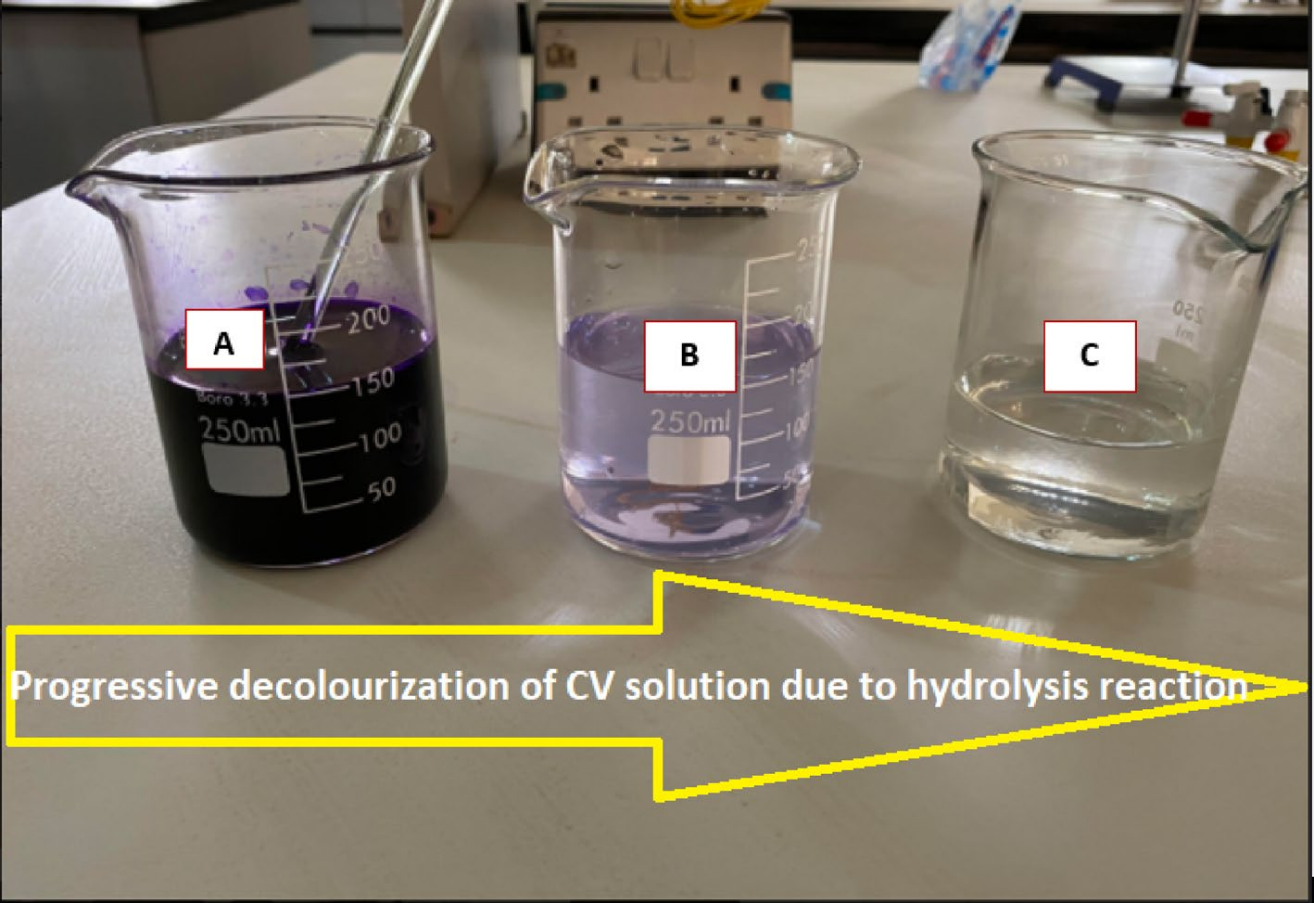
Image of the physical view of the decolourization due to hydrolysis reaction of CV. (**A**) Stock solution of CV (**B**) hydrolysis reaction in progress (**C**) hydrolysis reaction completed.

Runs of hydrolysis reaction at varying NaOH concentrations of 0.008, 0.016 and 0.024 M and constant initial CV concentration of 26.35 × ${10}^{-6}$ M were carried out at temperatures of 21 and 6 °C representing ambient temperature and temperature below ambient, respectively. The reactions were carried out in a 250 mL glass beaker used as a batch reactor. Progress of the reaction was monitored from the beginning by taking spectrophotometer absorbance at wavelength of 565 nm in intervals of 30 s until a total disappearance of colour was observed. Plate 1 shows the physical decolourization process involved in the hydrolysis reaction.

## Results and discussion

### Reaction kinetics

**Figure 2:**
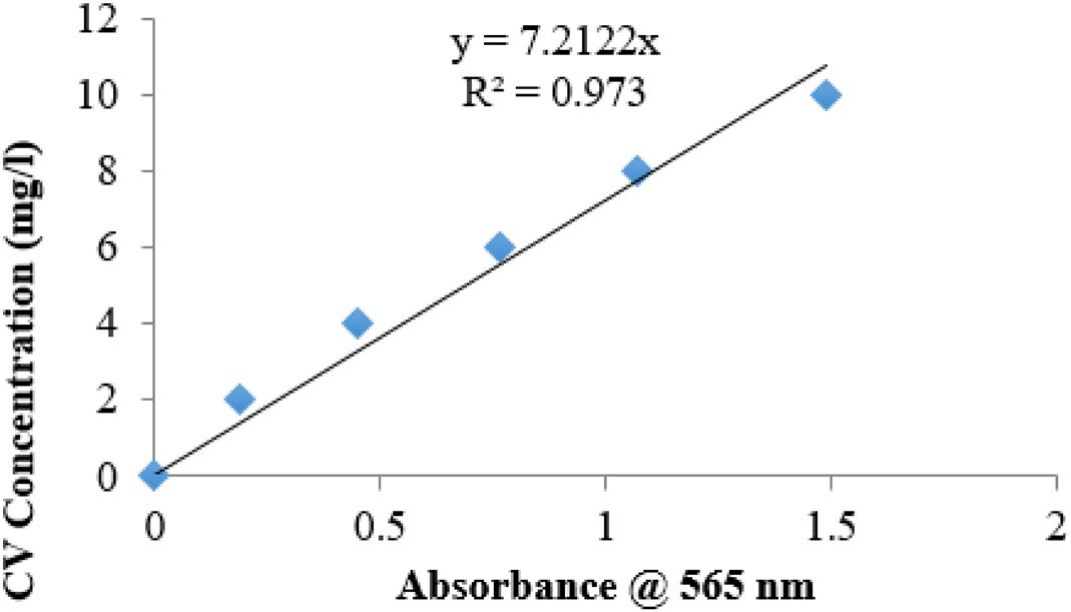
Calibration curve for the concentration of CV at varying spectrophotometer absorbance.

Figure 2 shows the calibration curve for the concentrations of CV against absorbance reading of the spectrophotometer during the hydrolysis reaction.

Using the straight line graph equation of Fig. 2, concentration of CV at any absorbance reading could be calculated using Eq. (9)

9 $${\text{C}} = {7}.{212} \times {\text{Absorbance}}$$

where C is concentration of CV.

**Figure 3:**
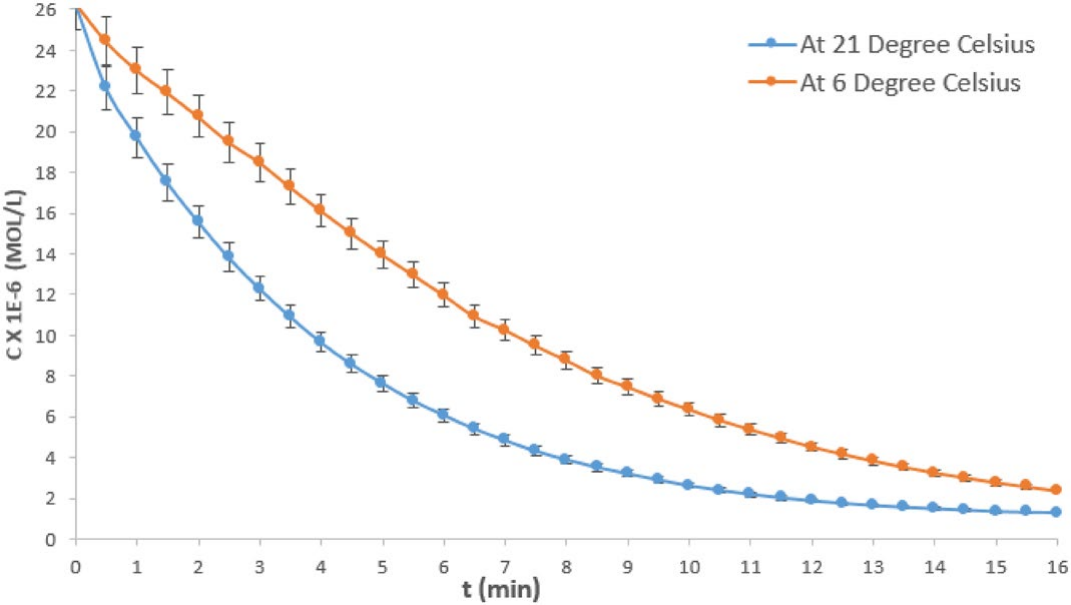
C–t curve for the hydrolysis of crystal violet at 0.016 M NaOH and variable temperature.

To determine the overall rate order of the hydrolysis reaction concentration–time date at an intermediate NaOH concentration was analysed. Figure 3 shows the concentration vs time (C–t) curves for the hydrolysis reaction of crystal violet at 6 and 21 °C, respectively using 0.016 M NaOH. The data was higly replicable, the percentage error obtained for four experimental repitition was 1.38%. Sodium hydroxide concentration of 0.016 M was selected being the intermediate concentration for this study. Typical of C–t graphs, it could be observed that the CV concentration depletes as the reaction time progresses for both curves, until the reaction was almost completed.

The generalized kinetic formula for nth order reaction^10^ can be written as

10 $${t}_{F}= \left(\frac{{F}^{1-n}-1}{k(n-1)}\right){C}_{Ao}^{1 - n}$$

where ${t}_{F}$ is time for F fractional disappearance of the reactant, k is reaction rate constant, ${C}_{Ao}$ is initial concentration of reactant A. In this case A is CV. Therefore we have; 

11 $$log\;{t}_{F}=log\;\left(\frac{{F}^{1-n}-1}{k(n-1)}\right)+\left(1-n\right)\;log\;{C}_{Ao}$$

Using the half-life approach, meaning taking the F factor as 0.5, we have;

12 $$log\;{t}_{0.5}=log\;\left(\frac{{0.5}^{1-n}-1}{k(n-1)}\right)+\left(1-n\right)\;log\;{C}_{Ao}$$

**Figure 4:**
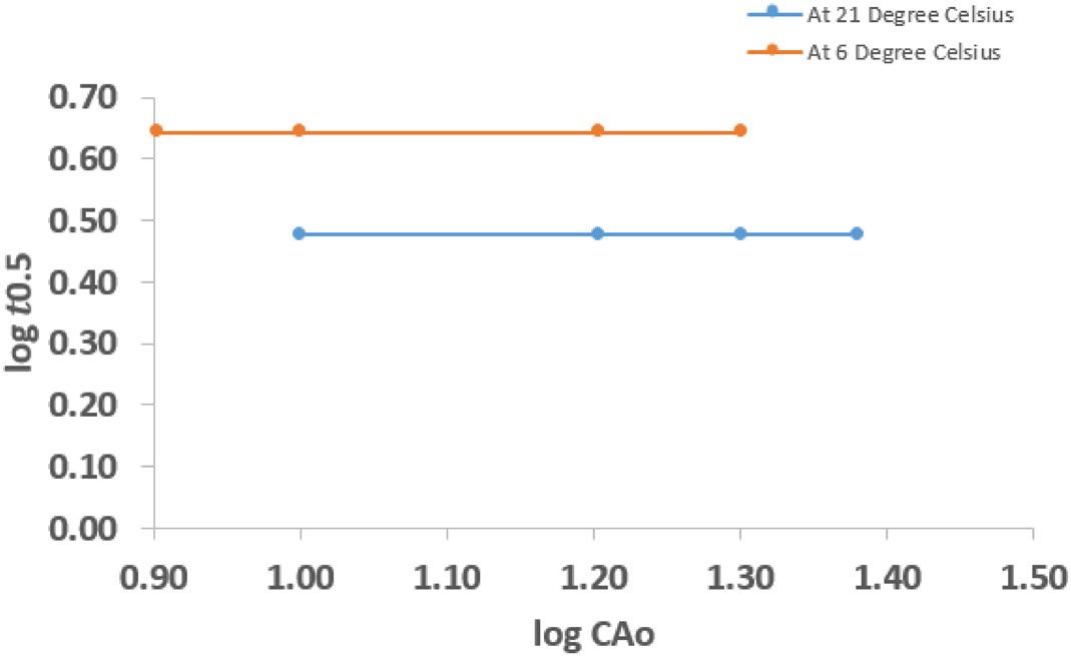
log ${t}_{0.5}$ vs log ${C}_{Ao}$ graph for hydrolysis of CV at 0.016 M NaOH and variable temperature.

Figure 4 shows plots of $log\;{t}_{0.5}$ and ${log\;C}_{Ao}$ for the various ${\mathrm{C}}_{\mathrm{Ao}}$ and ${t}_{0.5}$ derived from Fig. 3, for hydrolysis at 0.016 M NaOH, and variable temperature. At 21 °C it could be observed that the plot gives a straight horizontal line, implying that the slope of the graph is zero. Therefore, from Eq. (12), it can be deduced that;

13 $$\left( {{1}{-}{\text{n}}} \right) = {\text{slope}} = 0$$

14 $${\text{Therefore}},\quad {\text{n}} = {1}$$

Therefore, the hydrolysis reaction at 21 °C was 1st order reaction.

Similarly, at 6 °C the plot is a straight horizontal line, implying that the slope of the graph is zero. Hence, it also follows that the hydrolysis reaction at 6 °C was 1st order reaction. Therefore, it can be established that the overall rate order of hydrolysis of CV is a 1st order reaction at both ambient temperature and below ambient temperatures.

Generally, for 1st order reactions, Eq. (4) can be simplified as follows;

15 $$\frac{ - dC}{{dt}} = k^{\prime}{\text{C}}$$

16 $$\int\limits_{{C_{Ao} }}^{{C_{A} }} {\frac{ - dC}{C}} = \int\limits_{0}^{t} {k^{\prime}\;{\text{dt}}}$$

17 $$- ln\frac{{C_{A} }}{{C_{Ao} }} = k^{\prime}{\text{t}}$$

**Figure 5:**
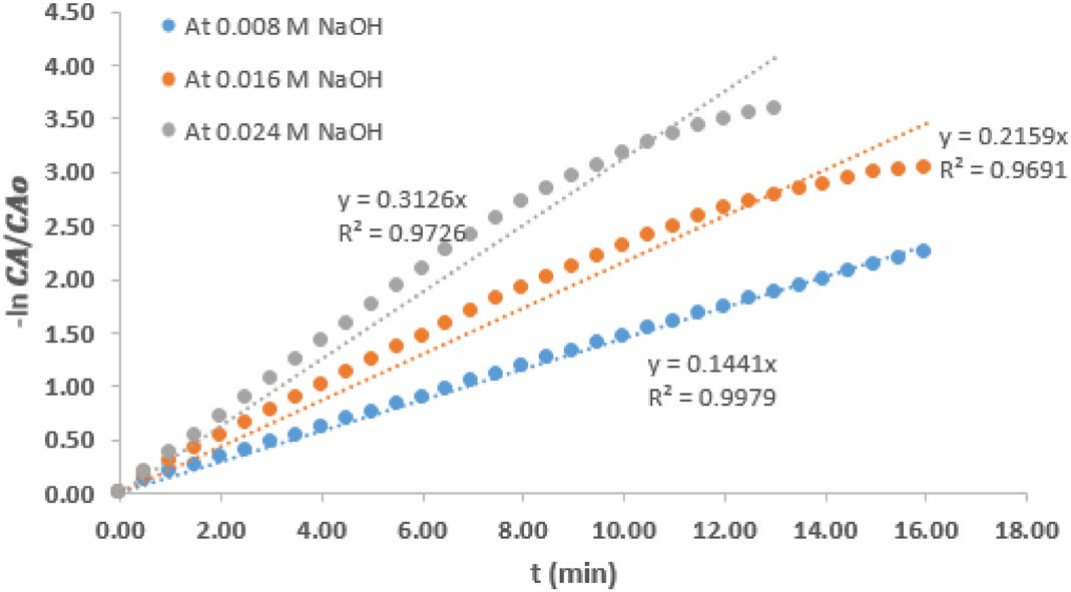
− ln $\frac{{C}_{A}}{{C}_{Ao}}$ vs time graph for hydrolysis of CV at 21 °C and variable concentration of NaOH.

Figure 5 shows plots of $-\mathrm{ln}\frac{{C}_{A}}{{C}_{Ao}}$ against time for the various NaOH concentrations for hydrolysis reaction carried out at 21 °C. The slope of straight-line graph obtained for each is equal to the pseudo rate constant $(k^{\prime})$ of the hydrolysis reaction at the various NaOH concentrations.

**Figure 6:**
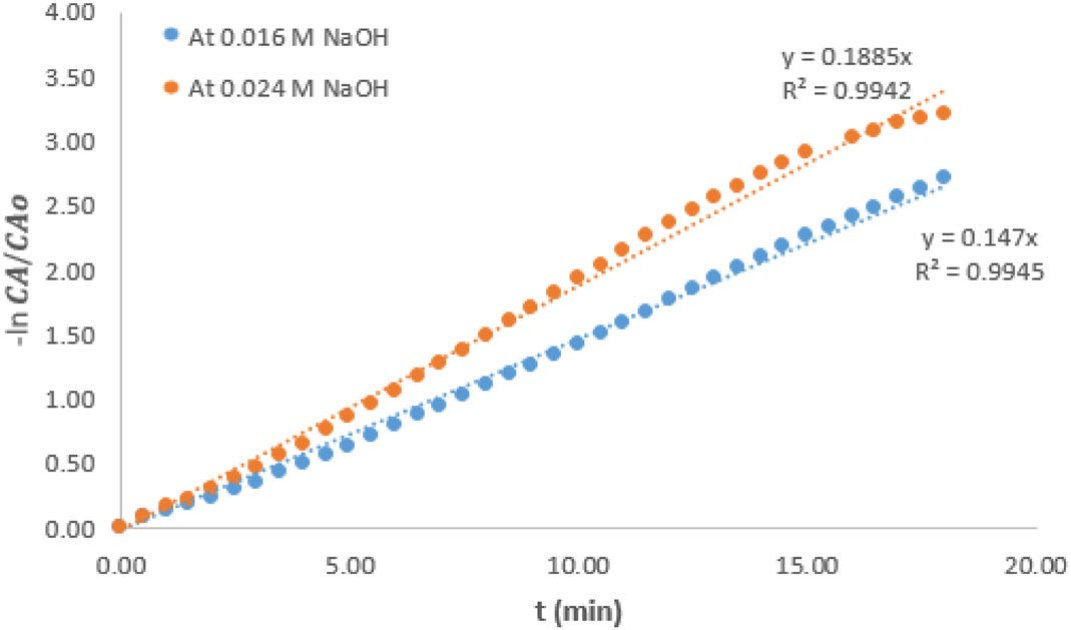
− ln $\frac{{C}_{A}}{{C}_{Ao}}$ vs time graph for hydrolysis of CV at 6 °C and variable concentration of NaOH.

Figure 6 shows plots of $-ln\frac{{C}_{A}}{{C}_{Ao}}$ against time for hydrolysis reaction carried out at 6 °C and NaOH concentrations of 0.016 and 0.024 M respectively.

Table 1 presents various values of pseudo rate constant of the hydrolysis reaction carried out at the two temperatures and variable concentration of NaOH. It could be observed that values of $k^{\prime}$ at a particular temperature increased as NaOH concentration increased. Considering the reaction condition at 21 °C and the intermediate concentration of 0.016 M, it could be observed that the $k^{\prime}$ value which was 0.230/min decreased by 36.5% when the NaOH concentration was reduced by 50%. However, $k^{\prime}$ value increased by 36% when the concentration was increased by 50%. At 6 °C reaction condition, the $k^{\prime}$ value at intermediate NaOH concentration of 0.016 M was 0.147/min. A similar trend was observed as the $k^{\prime}$ value was increased by 29% when the NaOH concentration increased by 50%. It can also be deduced that for any change in the NaOH concentration, the value of $k^{\prime}$ is affected proportionately. This observation is consistent with the report of other related work^11^ and this observation further shows that $k^{\prime}$ can only be pseudo rate constant, not the actual rate constant.

**Table 1:** Pseudo rate constant at variable reaction temperature and NaOH concentration.

Temperature (°C)	21	6
NaOH concentration (M)	$k^{\prime}$ (min^−1^)	$k^{\prime}$ (min^−1^ )
0.024	0.313	0.189
0.016	0.230	0.147
0.008	0.146	Not determined

Values of the actual reaction rate constants (k) at the various reaction conditions were obtained by applying Eq. (5). Therefore, values of $k^{\prime}$ obtained at each reaction condition and the corresponding NaOH concentration, [OH^−^] at that condition were substituted into Eq. (5). To obtain a solution, the rate order with respect to [OH^−^], *m*, was first determined using Eq. (8) and two set of [OH^−^] values were used at each reaction temperature as indicated in the derivation of Eqs. (8a) and (8b). [OH^−^] values of 0.024 and 0.016 M were used for this analysis. Hence, at reaction temperature of 21 °C, the rate order with respect to [OH^−^], *m* was determined as;

8a $$m = \frac{{log\frac{{ k_{1}^{\prime } { }}}{{k_{2}^{\prime } }}}}{{log\frac{{\left[ {{\text{OH}}^{ - } } \right]_{1} { }}}{{\left[ {{\text{OH}}^{ - } } \right]_{2} }}}} = \frac{{log\frac{{0.230{ }}}{0.313}}}{{log\frac{{0.016{ }}}{0.024}}} = 0.76$$

At reaction temperature of 6 °C the rate order with respect to [OH^−^], *m* was determined as;

8b $$m = \frac{{log\frac{{ k_{1}^{\prime } { }}}{{k_{2}^{\prime } }}}}{{log\frac{{\left[ {{\text{OH}}^{ - } } \right]_{1} { }}}{{\left[ {{\text{OH}}^{ - } } \right]_{2} }}}} = \frac{{log\frac{0.147}{{0.189}}}}{{log\frac{{0.016{ }}}{0.024}}} = 0.62$$

Therefore, substituting the corresponding values of [OH^−^], $k^{\prime}$ and *m* into Eq. (5) the actual reaction rate constants (k) at the various reaction temperatures were determined as presented in Table 2.

**Table 2:** Actual rate constant at variable reaction temperature and NaOH concentration.

Temperature (K)	294	279
NaOH concentration (M)	${\text k}\left[\left( {\frac{{{\text{mol}}}}{{\text{L}}}} \right)^{{ - 0.9}} min^{-1}\right]$	${\text k}\left[\left( {\frac{{{\text{mol}}}}{{\text{L}}}} \right)^{{ - 0.9}} min^{-1}\right]$
0.024	7.2	1.9
0.016	7.2	1.9

The rate law of the reaction can be stated using Eq. (3). It could be observed from Table 2 that irrespective of the concentration of the NaOH the rate constant of the hydrolysis reaction at 21 °C was 7.2 $\left( {\frac{{{\text{mol}}}}{{\text{L}}}} \right)^{{ - 0.9}} min^{-1}$. It would be recalled that the overall order of the reaction was determined as 1st order, therefore, x = 1 − m. Using value of *m* = 0.76 from Eq. (8a), hence, at reaction temperature of 21 °C we have x = 1 − 0.76 = 0.24. Therefore, the reaction rate law at 21 °C is given by Eq. (18).

18 $$- r_{A} = kC_{A}^{x} \left[ {OH^{ - } } \right]^{m} = 7.2\left( {\frac{{{\text{mol}}}}{{\text{L}}}} \right)^{{ - 0.9}} min^{-1}\;C_{A}^{0.24} \left[ {OH^{ - } } \right]^{0.76}$$

It could be observed from Table 2 that irrespective of the concentration of the NaOH the rate constant of the hydrolysis reaction at 6 °C was 1.9 $$\left( {\frac{{{\text{mol}}}}{{\text{L}}}} \right)^{{ - 0.9}} min^{-1}$$$$.$$ Similarly, using value of *m* = 0.62 from Eq. (8b), at reaction temperature of 6 °C we have x = 1 − 0.62 = 0.38. Therefore, the reaction rate law at 6 °C is given by Eq. (19)

19 $$- r_{A} = kC_{A}^{x} \left[ {OH^{ - } } \right]^{m} = 1.9\left( {\frac{{{\text{mol}}}}{{\text{L}}}} \right)^{{ - 0.9}} min^{-1}\;C_{A}^{0.38} \left[ {OH^{ - } } \right]^{0.62}$$

### Reaction thermodynamics

Equation (20) is the Arrhenius relationship for determination of activation energy (${E}_{a}$) of the hydrolysis reaction^10^

20 $$ln\frac{{k}_{2}}{{k}_{1}}= \frac{{E}_{a}}{R} \left(\frac{1}{{T}_{1}} - \frac{1}{{T}_{2}}\right)$$

where R = 8.314 J/mol-K

It could be observed from the results in Table 2 that values of the rate constant, k, for the hydrolysis reaction is only temperature dependent. The rate constant value is independent of the NaOH concentration as the value at a particular temperature remain constant irrespective of the concentration. This is consistent with literature information about such reaction^10^. Solutions of Eq. (20) using values of the variables in Table 2 gives

$$E_{a} = \frac{{T_{1} T_{2} R\;{\text{ln}}\;\frac{{k_{2} }}{{k_{1} }}}}{{T_{2} - T_{1} }} = \frac{{279 \times 294 \times 8.314\;{\text{ln}}\;\frac{7.2}{{1.9}}}}{294 - 279} = 60.57\;{\text{kJ/mol}}$$

Equation (21) is the thermodynamic relation for the enthalpy of the reaction and the activation energy^12^

21 $$\Delta {H}_{a}={E}_{a}-RT$$

where $\Delta {H}_{a}$ is the enthalpy at activation.

Hence, using Eq. (21) and the calculated activation energy, the enthalpy of hydrolysis reaction of crystal violet at 21 °C was determined as

$$\Delta H_{a} = 60,569{-}8.314 \times 294 = 58.13\;{\text{kJ/mol}}$$

Similarly, the enthalpy of hydrolysis reaction at 6 °C was determined as

$$\Delta H_{a} = 60,569{-}8.314 \times 279 = 58.25\;{\text{kJ/mol}}$$

Equation (22) is the Eyring equation^11,13^ which relates the reaction rate constant, (k) with the enthalpy (∆H) and entropy (∆S)

22 $$k= \frac{{Tk}_{B}}{h}{e}^{-\frac{\Delta H}{RT}}{ e}^{\frac{\Delta S}{R}}$$

where ${k}_{B}$ is the Boltzman’s constant = 1.381 × 10^–23^ J/K, *h* is the Planck’s constant = 6.626 × 10^–34^ Js, T is the absolute temperature in Kelvin and R is the gas constant.

To determine the entropy of the reaction, Eq. (22) can be re-written as Eq. (23)

23 $$\Delta \mathrm{S}=\left(\frac{\Delta H}{RT}+\mathrm{ln}\left(\frac{kh}{T{k}_{B}}\right)\right)R$$

At temperature of 21 °C (294 K), the rate constant (in per s) is k = 0.12 $\left( {\frac{{{\text{mol}}}}{{\text{L}}}} \right)^{{ - 0.9}} s^{-1}$. Therefore, we have

$$\Delta {\text{S}} = \left( {\frac{\Delta H}{{RT}} + \ln \left( {\frac{kh}{{Tk_{B} }}} \right)} \right)R = \left( {\frac{58125}{{8.314 \times 294}} + \ln \left( {\frac{{0.12 \times 6.626 \times 10^{ - 34} }}{{294 \times 1.381 \times 10^{ - 23} }}} \right)} \right) \times { }8.314 = - 64.72\;{\text{J/mol}}\;{\text{K}}$$

At temperature of 6 °C (279 K), the rate constant (in per s) is k = 0.032 $\left( {\frac{{{\text{mol}}}}{{\text{L}}}} \right)^{{ - 0.9}} s^{-1}$. Therefore, we have;

$$\Delta {\text{S}} = \left( {\frac{\Delta H}{{RT}} + \ln \left( {\frac{kh}{{Tk_{B} }}} \right)} \right)R = \left( {\frac{58249}{{8.314 \times 279}} + \ln \left( {\frac{{0.032 \times 6.626 \times 10^{ - 34} }}{{279 \times 1.381 \times 10^{ - 23} }}} \right)} \right) \times 8.314 = - 64.29\;{\text{J/mol}}\;{\text{K}}$$

Equation (24) is the thermodynamic relation for the entropy, enthalpy and Gibbs free energy (∆G)

24 $$\Delta {\text{G}} = \Delta {\text{H}}{-}{\text{T}}\Delta {\text{S}}$$

Substituting values of ΔH and ΔS at the various temperatures, we have ΔG values at 21 and 6 °C as 77.15 and 76.19 kJ/K, respectively.

It could be observed that the hydrolysis reaction of crystal violet is an endothermic reaction having enthalpies of 58.13 and 58.25 kJ/mol at temperatures of 21 and 6 °C, respectively. At 21 °C the entropy and Gibbs free energy of the hydrolysis reaction were − 64.72 J/mol K and 77.15 kJ/K, respectively. While at 6 °C the entropy and Gibbs free energy of the reaction were − 64.29 J/mol K and 76.19 kJ/K, respectively. In comparison with other reported works^11^; this study was carried out at temperatures of 21 and 6 °C, constant initial CV concentration, [CV] of 2.6 × ${10}^{-5}$ M, and varying NaOH concentrations, [OH] of 0.008–0.024 M. Felix^11^ reported similar study at higher temperatures of 25 and 40 °C, similar constant initial CV concentration, [CV] of 1.00 × 10^−5^ M and varying low NaOH concentrations, [OH^−^] of 6.67 × 10^−4^ to 3.35 × 10^−3^ M. The activation energy reported by Felix^11^ was 15.60 kJ/mol which is 74% less than the activation energy value of 60.57 kJ/mol determined in this study. At ambient temperature, the entropy of reaction, enthalpy and Gibbs free energy reported by Felix^11^ were − 260 J/mol K, 13.95 kJ/mol and 91.43 kJ/mol, respectively. Whereas this study obtained − 64.29 J/mol K, 58.13 kJ/mol and 76.19 kJ/K as the entropy, enthalpy and Gibbs free energy, respectively. Although, the enthalpy reported by Felix^11^ was 76% less than the enthalpy value of this study, both works corroborate that the hydrolysis of CV is endothermic as can be deduced from the positive enthalpy values in both. Furthermore, both works corroborate that the reaction was non-spontaneous at all temperature as can be deduced from the negative entropy values in both. The lower thermodynamic parameters reported by Felix^11^ were due to the lower NaOH concentration used which was averagely 92% lower than the NaOH concentration used in this work. The low NaOH concentration tends to make the reaction less spontaneous. As for the kinetic parameters other reported works^7,8,13^ also corroborate that the overall rate order of the hydrolysis of CV is 1st order. However, the previous works neither reported values of the individual rate order with respect to CV and NaOH nor did they report the detailed kinetic rate law for the hydrolysis reaction of CV, this information are the major novelty of this work.

## Conclusion

Hydrolysis reaction of crystal violet is an endothermic reaction which is insensitive to temperature change, the enthalpy, entropy and Gibbs free energy obtained at ambient temperature were 58.13 kJ/mol, − 64.72 J/mol K and 77.15 kJ/K, respectively. Hence, the reaction was non-spontaneous at all temperature. The reaction was insensitive to temperature change as 15 °C drop in temperature from ambient condition only decreased the enthalpy of the reaction by 0.2% and increased the entropy and Gibbs free energy by 0.7% and 1.3%, respectively. The activation energy of the reaction was determined as 60.57 kJ/mol at both ambient and below ambient temperatures. Kinetic analysis of the hydrolysis reaction indicated that the overall rate order of the reaction was first order having rate constant values of 7.2 and 1.9 $\left( {\frac{{{\text{mol}}}}{{\text{L}}}} \right)^{{ - 0.9}} min^{-1}$ at ambient temperature and below ambient temperature, respectively. The rate constant of the reaction was independent of the NaOH concentration. This study has also shown that the individual rate order with respect to NaOH and CV were temperature dependent. At ambient temperature the rate constant with respect to NaOH and CV were determined as 0.24th and 0.76th, respectively. Below ambient temperature the rate constant with respect to NaOH and CV were determined as 0.38th and 0.62th, respectively. Kinetic rate law of hydrolysis reaction of crystal violet carried out at ambient temperature of 21 °C was determined as

$$- r_{A} = 7.2\left( {\frac{{{\text{mol}}}}{{\text{L}}}} \right)^{{ - 0.9}} min^{-1}\;C_{A}^{0.24} \left[ {OH^{ - } } \right]^{0.76}$$

The rate law of the reaction carried out below ambient temperature at 6 °C was determined as

$$- r_{A} = 1.9\left( {\frac{{{\text{mol}}}}{{\text{L}}}} \right)^{{ - 0.9}} min^{-1}\;C_{A}^{0.38} \left[ {OH^{ - } } \right]^{0.62}$$

At ambient temperature of 21 °C the rate of the reaction was 5.3 times dependent on the concentration of NaOH than CV. At temperature of 6 °C the rate of the reaction was 1.6 times dependent on the concentration of NaOH than CV. Generally, it could be deduced that the concentration of NaOH largely determines the rate of hydrolysis of crystal violet with the dependency on NaOH concentration reducing as the reaction temperature drops from ambient condition. Therefore, our initial hypothesis that [CV] is the rate limiting reactant is justified.
